# Supplementary material for: Immunostimulatory Activity of Cordyceps militaris Fermented with Pediococcus pentosaceus SC11 Isolated from a Salted Small Octopus in Cyclophosphamide-Induced Immunocompromised Mice and Its Inhibitory Activity against SARS-CoV 3CL Protease
Source: Microorganisms. 2022 Nov 23;10(12):2321. doi: 10.3390/microorganisms10122321 (PMC9781638; doi:10.3390/microorganisms10122321)
Supplement: Supplementary file 1 [file microorganisms-10-02321-s001.zip › microorganisms-1915879-supplementary.pdf]

Supplementary data

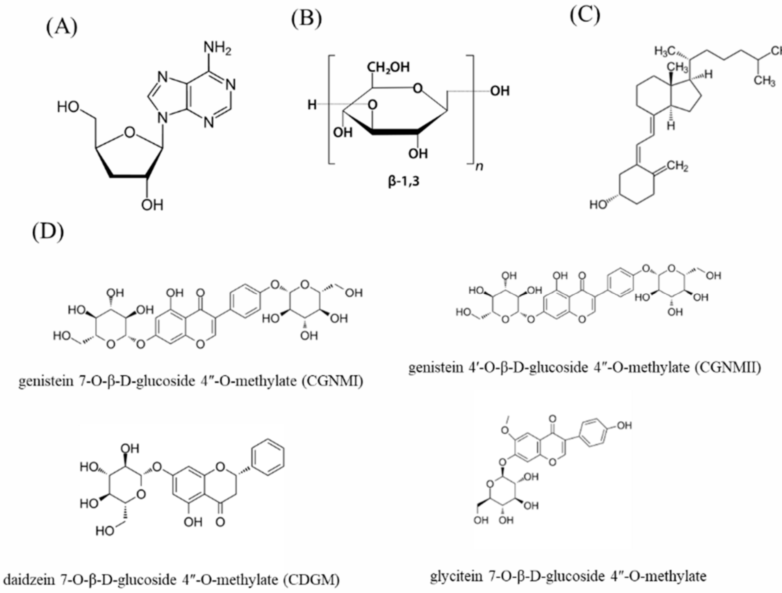

Supplementary Figure S1. The structure of potential bioactive compounds in GRC-SC11. (A) Cordycepin. (B) Beta glucan. (C) vitamin D. (D) novel isoflavone methylglycosides.
